# Optimisation of Mechanical Properties in Saw-Dust/Woven-Jute Fibre/Polyester Structural Composites under Liquid Nitrogen Environment Using Response Surface Methodology

**DOI:** 10.3390/polym13152471

**Published:** 2021-07-27

**Authors:** Velmurugan Ganesan, Vigneshwaran Shanmugam, Babu Kaliyamoorthy, Sekar Sanjeevi, Suresh Kumar Shanmugam, Vasudevan Alagumalai, Yoganandam Krishnamoorthy, Michael Försth, Gabriel Sas, Seyed Mohammad Javad Razavi, Oisik Das

**Affiliations:** 1 Department of Agricultural Engineering, Saveetha School of Engineering, Saveetha Institute of Medical and Technical Sciences, Chennai 602105, India; 2 Department of Mechanical Engineering, Saveetha School of Engineering, Saveetha Institute of Medical and Technical Sciences, Chennai 602105, India; s.vigneshwaren@gmail.com (V.S.); vasudevana.sse@saveetha.com (V.A.); 3 Department of Mechanical Engineering, Sri Sivasubramaniya Nadar College of Engineering, Chennai 603110, India; babuk@ssn.edu.in; 4 Department of Mechanical Engineering, Hindusthan Institute of Technology, Coimbatore 641028, India; yessekar007@gmail.com; 5 Faculty of Mechanical Engineering, Kalasalingam Academy of Research and Education, Krishnankoil 626128, India; sureshme48@gmail.com; 6 Department of Mechanical Engineering, ARM College of Engineering and Technology, Chennai 602105, India; yogamech89@gmail.com; 7 Structural and Fire Engineering Division, Department of Civil, Environmental and Natural Resources Engineering, Luleå University of Technology, 97187 Luleå, Sweden; michael.forsth@ltu.se (M.F.); gabriel.sas@ltu.se (G.S.); 8 Department of Mechanical Engineering, Norwegian University of Science and Technology, 7491 Trondheim, Norway

**Keywords:** jute, structural composites, Taguchi, response-surface methodology

## Abstract

Natural fibre-based composites are replacing traditional materials in a wide range of structural applications that are used in different environments. Natural fibres suffer from thermal shocks, which affects the use of these composites in cold environment. Considering these, a goal was set in the present research to investigate the impact of cryogenic conditions on natural fibre composites. Composites were developed using polyester as matrix and jute-fibre and waste Teak saw-dust as reinforcement and filler, respectively. The effects of six parameters, viz., density of saw-dust, weight ratio of saw-dust, grade of woven-jute, number of jute layers, duration of cryogenic treatment of composite and duration of alkaline treatment of fibres on the mechanical properties of the composite was evaluated with an objective to maximise hardness, tensile, impact and flexural strengths. Taguchi method was used to design the experiments and response-surface methodology was used to model, predict and plot interactive surface plots. Results indicated that the duration of cryogenic treatment had a significant effect on mechanical properties, which was better only up to 60 min. The models were found to be statistically significant. The study concluded that saw-dust of density 300 kg/m^3^ used as a filler with a weight ratio of 13 wt.% and a reinforcement of a single layer of woven-jute-fibre mat of grade 250 gsm subjected to alkaline treatment for 4 h in a composite that has undergone 45 min of cryogenic treatment presented an improvement of 64% in impact strength, ca. 21% in flexural strength, ca. 158% in tensile strength and ca. 28% in hardness.

## 1. Introduction

Growing concerns for the environment have accelerated the replacement of synthetic composites and plastics with natural fibre composites (NFC), which potentially have lower carbon footprint. Natural fibre-based composites have also been found in structural applications. For example, the use of jute-based natural fibre composites in structural applications is recommended due to the improved strain behaviour and fatigue strength [1]. More specifically, composites can be used to store cryogenic liquids in space shuttles or satellites (liquefied oxygen and hydrogen) [2]. Structural composites are also used in extremely cold weather. In these circumstances, the temperature variation causes high thermal stress in materials, which lead to failure [3]. The hydrophilic nature (presence of free and bound water in the organic biomass) of natural fibre composites is a critical factor that limits the use of natural fibre composites in the cryogenic conditions. Natural fibres’ hydrophilic nature (i.e., higher volume of ice than that of water) causes poor load transfer and unexpected failure in cold environments. To avoid such a failure, the composite strength must be increased to withstand thermal stress under cryogenic conditions [4]. Hybridisation and chemical treatment of natural fibres are the notable methods that are in practice to increase the strength of such composites.

India is the top jute-producing nation in the world, contributing over 50% of the global jute production [5]. Such an abundant availability of jute fibre could be used for manufacturing bio-based composite materials. Jute possesses greater stiffness and strength compared to other natural fibres [6]. Stable compounds could be obtained owing to the bidirectional nature of jute fibres that could offer resistance to cracking. Apart from being renewable and easily available, the motivation for employing jute fibre instead of conventional glass fibres arises from the fact that it has lower specific gravity (1.45 GPa) and workable specific modulus (19 GPa) when compared to that of glass fibres (2.56 GPa and 29 GPa, respectively) [7]. Gowda et al. [8] conducted an experimental study on untreated woven-jute reinforced polyester composites and demonstrated its potential use in numerous consumer products. Vinod et al. [9] investigated the thermo-mechanical characterisation of *Calotropis gigantea* stem powder-filled jute fibre-reinforced epoxy composites and showed that the higher weight percentage *Calotropis gigantea* filled jute fibre composites showed superior results in tensile, flexural, compression, hardness, and impact properties than the ones with partially filled and no filler materials. Tavassolli et al. [10] showed that hydrothermally treated wood fibres showed improvement in mechanical properties. Hence, a renewable filler material like saw-dust could be dispersed in resin and improvement in properties could be achieved. Pinto et al.’s [11] investigation demonstrated the efficacy of jute epoxy composites for structural applications. The treated-jute/epoxy composites showed enhanced toughness and interlaminar shear strength. According to the findings of the Assis et al. [12] investigation, the use of jute fibre in the multilayer armour system (MAS) can reduce the weight of the MAS by 5.4% and the cost by 474%. The jute/kenaf-based hybrid composites showed enhanced performance in the low velocity impact test. The hybrid jute/kenaf composites withstood the 30 J impact energy without being penetrated. Elsewhere, Park et al. [13] evaluated the mechanical properties of single jute fibres after alkaline, silane and thermal treatments using uni- and bi-modal Weibull distribution. They showed that alkaline- and silane-treated jute fibres showed an increase in the mechanical properties of single jute fibres, while thermal treatment resulted in deterioration of mechanical properties. Rafiquzzaman et al. [14] asserted that jute fibre could serve as a partial replacement for glass-fibres for light load structural applications. Based on the aforementioned findings from literature, this study used jute-fibre with saw-dust as a filler material dispersed in polyester matrix. Saw-dust could be used a filler material because of its renewable nature, and its availability as waste material from sawmills at a cheap cost [2]. The fibres were chemically treated, and the final fabricated Fibre-Reinforced Composites (FRC) were then subjected to cryogenic treatment to improve the mechanical properties.

Alkaline treatment, also known as mercerisation, disrupts the hydrogen bonding in the molecular structure of the fibre and increases the surface roughness. This improves the interfacial adhesion between the matrix and the fibre. This treatment also eliminates lignin, waxy and oily materials that cover the external wall of the fibre [15]. Ray et al. [16] achieved an improvement in crystallinity of jute fibres by treating it to 5% alkali solution for up to 8 h. Enhancement in modulus, tenacity, flexural strength and laminar shear strength was reported after alkali treatment. Rajesh and Prasad [17] showed that jute fibres treated with 10% NaOH improved the tensile strength of the composites. They demonstrated that the alkali-treated (5%, 10%, and 15%) fibre had considerably increased the tensile strength of the composites compared to its untreated counterpart.

The mechanical properties of the fibre-reinforced composites could be modified on the basis of requirements by altering the fibre–resin–filler combination [18]. The renewable, cheap, non-abrasive nature of jute-fibre makes it an attractive option for use as reinforcement in composites. However, its hydrophilic nature can create binding issues with commercial synthetic resins resulting in poor strength and stiffness. Furthermore, composites can have a wide range of applications in aerospace industry and as structural components in colder climates where extreme temperature variations are possible. Therefore, an attempt was made to overcome these limitations by chemical treatment and hybridisation. The present study employed a modelling and optimisation approach, which can be used to study the interactive effects of the considered parameters and to quantify the individual influences of these parameters on different mechanical properties of the composite. This study used six parameters, viz., density of saw-dust, weight ratio of saw-dust, grade of woven-jute, number of jute layers, duration of cryogenic treatment of composite and duration of alkaline treatment of jute-fibre for optimization with an objective to maximise tensile strength, impact strength, flexural strength and hardness. These six parameters were considered, since these are the major influencing factors that greatly affect natural fibre composites’ properties [19]. The novel aspect of the current study can be highlighted by the fact that this is one of the first studies to investigate composites with woven jute as reinforcement and teak saw-dust as filler material in polyester resin under cryogenic conditions. It is envisaged that the results of this investigation could open up new application routes for natural fibre-based composites in extreme cold environments. Moreover, the effects of cryogenic treatment on the composite and alkaline treatment of jute-fibre on mechanical properties were investigated together for the first time. The main objective of this study is to identify the most significant parameters that have the highest effect on the mechanical properties of natural fibre composites under cryogenic conditions. The specific aims of the study can be sub-divided as follows: (i) to identify the statistically significant parameters that affect the mechanical properties of the fabricated composite, (ii) to develop regression models for the mechanical properties using RSM, (iii) to predict the mechanical properties, e.g., tensile strength, impact strength, flexural strength and hardness of the composite, and (iii) to determine an optimum combination of the considered factors to achieve the best possible performance properties.

## 2. Materials and Methods

### 2.1. Materials

**Figure 1:**
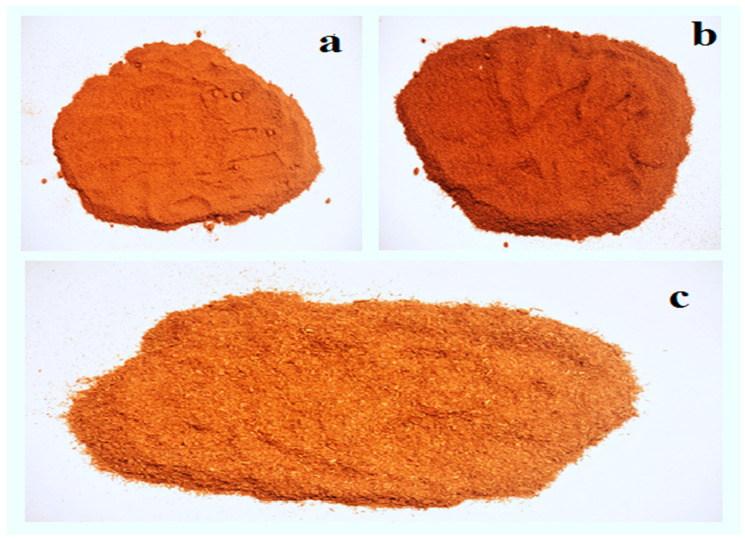
Saw-dust of weight densities (**a**) 200 kg/m^3^ (**b**) 250 kg/m^3^ and (**c**) 300 kg/m^3^.

**Figure 2:**
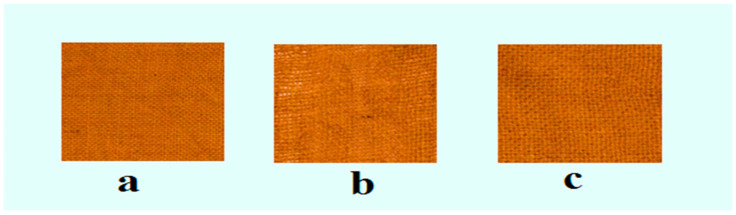
Woven-jute fibre of grades (**a**) 250 gsm, (**b**) 300 gsm and (**c**) 350 gsm.

Teak saw-dust from miscellaneous wooden logs was collected from a saw-mill in Madurai, Tamil Nadu, India. Woven jute mats were collected from a Jute service centre in Madurai, Tamil Nadu, India. Both saw-dust and woven jute were sun-dried for 2 days to remove moisture. Saw-dust is an aggregate of mixed powder of different grain sizes and was sorted into three types based on the density (200 kg/m^3^, 250 kg/m^3^ and 300 kg/m^3^) using sieves as shown in Figure 1. The jute fibres were separated based on its gsm values (250 gsm, 300 gsm and 350 gsm) as shown in Figure 2. Unsaturated polyester (resin), methyl-ethyl ketone peroxide (catalyst) and cobalt naphthenate (accelerator) were procured from GVR enterprises, Madurai, Tamil Nadu, India.

### 2.2. Preparation of Saw-Dust and Jute Fibres

Saw-dust and jute fibres were individually washed with 1 to 2% detersive solvents at 60 to 70 °C for 1 h to remove any impurities, followed by rinsing with distilled water and finally dried in a vacuum oven at 70 °C for 1 h and 30 min. The dried fibres were designated as untreated fibres. The dried saw-dust was used as fillers. The jute fibres were then chemically treated. During the treatment, the jute fibres were first de-waxed by soaking in 2:1 mixture of benzene and ethanol for 70 to 72 h at 50 °C, and then the fibres were thoroughly washed with distilled water and dried for 24 h. The de-waxed yarns were then plunged in a beaker containing 5% NaOH solution. According to the experimental design, the duration of alkali treatment was varied for 2 h, 4 h and 6 h. Three sets of fibres were prepared in this fashion. They were then washed thoroughly using distilled water and were air-dried for 12 h. Finally, they were kept in an oven at 50 °C for 5 h.

### 2.3. Fabrication

A stainless-steel mould of dimensions 300 mm × 300 mm × 3 mm was used. Unsaturated polyester resin was combined with 1% by wt. of cobalt naphthenate & 1% by wt. of methyl-ethyl ketone peroxide and was mixed thoroughly by stirring. The treated saw-dust along with the woven jute fibres were used for composite fabrication by hand lay-up technique. Saw-dust of the different weight fractions was first dispersed in the prepared polyester resin by intense stirring. The prepared resin mixture was poured inside the mould and spread out by using a hand roller. Woven jute-fibre was laid over the resin mixture and another layer of polyester resin was again poured and spread out using a hand-roller. Composites were prepared with up to three layers of woven-jute fibre sandwiched between the polyester resin matrices as per the experimental design requirements. The mould was then secured by bolting and then a uniform pressure of 50 kg/cm^2^ was applied over the mould and was allowed to cure at room temperature for 24 h. The fabricated hybrid composite samples were then kept in desiccators to prevent absorption of moisture.

Cryogenic treatment was carried out in a programmable temperature controlled cryogenic chamber. The temperature was brought down to −196 °C by controlled rate of cooling (3 °C/min). The fabricated samples were then immersed in liquid N_2_ at 77 K for cryogenic treatment for different durations (30 min, 60 min and 90 min) as per the experimental design. After the treatment, the composites were brought back to room temperature by controlled constant rate of heating of 40 °C/h.

### 2.4. Characterization

Microscopic examinations were carried out using a Zeiss SUPRA 55-VP scanning electron microscope (SEM) (Sathyabama Institute of Science and Technology, Chennai, India). The sample was sputter coated with 10 nm gold and several samples were examined to ascertain the observed phenomena. The micrographs were taken at the voltage of 30 kV and working distance was varied between 25 to 45 mm.

### 2.5. Parameters and Their Levels

Table 1 lists the parameters affecting the mechanical properties of the composites that were considered in this research with their levels. Chung and Greener demonstrated that filler concentration plays a prominent role in determining the properties of composite resins [20]. A good interfacial interaction between the resin matrix and filler material improves the performance of the composites. Hence, density of saw-dust and weight ratio of saw-dust were used as controllable parameters. Research has shown that fibre loading leads to better mechanical properties of the composites [21], and therefore, the grades of woven jute fibre and number of jute layers were considered as factors. Additionally, chemically treated natural fibres and cryogenically treated composites also have shown improvement in performance [22].

### 2.6. Experimental Design Matrix

This study employs an experimental matrix based on Taguchi L_27_ orthogonal array, which requires only 27 trials to evaluate the influence of considered parameters on measured responses as shown in Table 2. This saves times and cost as it would otherwise require 3^6^ = 729 trials for a 6-factor × 3-level full factorial experimentation.

### 2.7. Testing

The specimens were cut to the dimensions according to ASTM D3039 (25.4 mm wide and 250 mm long) for tensile testing, ASTM D790 (10 mm wide and 125 mm length) for flexural and ASTM D256 (12.7 mm wide and 64 mm length) for Izod impact testing. Specimens were tested in a Universal Test Machine (UTM) of capacity 5 kN (FIE UNITEK 9400 Series) (Indira Gandhi Centre for Atomic Research, Chennai, India). Loading was applied at the rate of 2 mm/min for testing. For flexural testing, the specimens were loaded for three-point bending with span/depth ratio (L/D) of 16:1. The tests were carried out in the same machine by applying 10 kN load at the rate of 2.8 mm/min. Impact testing was done in Izod testing machine (FIE, IT-30 Series). The flexural and impact strength were calculated using Equations (1) and (2), respectively. The values of these experimental tests are tabulated in Table 3.

Flexural strength (σ) = 3 PL/2 bd^2^ (1)

where σ is flexural strength, P is applied load at the fracture point, L is the length of the support span, b is width of the specimen, d is thickness of the specimen.

Impact Strength (IS) = (Observed Energy)/(Cross sectional Area)(2)

The hardness of the composites was measured using the Brinell hardness test technique according to the ASTM E10 protocol. Despite the number of experiments being significantly reduced, the total number of required experiments were still high (total of 54 experiments for samples treated with and without NaOH and cryogenic environment). Hence, single specimens were tested for each experiment and the raw data is provided in the supplementary information (S.I.) file.

### 2.8. Response-Surface Methodology

Response-surface methodology was used for regression modelling, generating response-surface plots and graphical analysis of the measured data. The experimental data derived from Table 6 were analysed using third-order polynomial models that were developed using Equation (3),

(3) $$Z=\beta_{o}+\overset{3}{\underset{i=1}{\sum }}\beta_{i}X_{i}+\overset{3}{\underset{i-1}{\sum }}\beta_{ii}X_{i}^{2}+\overset{n}{\underset{i<1}{\sum }}\beta_{ij}X_{i}X_{j}+\varepsilon$$

where *Z* is the response, *X_i_* are numeric values of the factors, terms *β*_0_, *β_i_*, *β_ii_* and *β_ij_* are regression coefficients, *i* and *j* are linear and quadratic coefficients, and *ε* is the experimental error [23]. The developed model equation would represent a correlation between the parameters and measured responses. Response-surface plots were constructed using these fitted models.

### 2.9. Desirability Approach

The optimal combination of density of saw-dust, weight ratio of saw-dust, grade of woven-jute, number of jute layers, duration of cryogenic treatment of the composite and duration of alkaline treatment of jute fibre can be obtained by incorporating desirability function with RSM. This approach is a statistical technique, which combines multiple responses like tensile strength, flexural strength, impact strength and hardness into a single dimensionless number called the desirability function. This technique involves transforming each measured response, *Z_i_* that varies over the range, 0 < *d_i_* < 1, where *d_i_* value indicates the individual desirability of response *Z_i_*. A value of 1 specifies a completely desirable response while a value of 0 specifies a completely undesirable response. The objective for each response is to ‘minimise’, ‘maximise’, ‘target’, ‘in range’ or ‘equal to’ based on the nature of the optimisation. In the present work, the objective is to simultaneously maximise all the tested mechanical properties. For this objective, the individual desirability, *d_i_* is defined by Equation (4) as

(4) $$d_{i}=0,\;\mathrm{when}\;Z_{i}\leq Low_{i}\;d_{i}={\left(\frac{Z_{i}-\;Low_{i}}{High_{i}-Low_{i}}\right)}^{t_{i}},\;\mathrm{when}\;Low_{i}<Z_{i}<High_{i}\;d_{i}=1,\;\mathrm{when}\;Z_{i}\;\geq High_{i}$$

where *Z_i_* is the value of the ith response, high and low represent the upper and lower limits of the response, respectively [23]. The weight *t_i_* ranges between 0.1 and 10 (*t_i_* > 1 implies greater emphasis to the chosen objective). Individual desirabilities of all responses were then added together by geometric mean to arrive at an overall desirability function, D, which again varies between 0 and 1, and it is calculated by Equation (5),

(5) $$D={(\prod_{i=1}^{n}d_{i}{}^{r_{i}})}^{\frac{1}{\sum r_{i}}}$$

where *r* is the importance assigned to a response with respect to the other responses. Importance varies from the most important (5) to the least important (l). The highest value of D presents a desirable and optimal solution. The top solutions based on the desirability function are then validated by performing confirmatory tests based on the set criterion.

## 3. Results

### 3.1. Cryogenic Treatment

**Figure 3:**
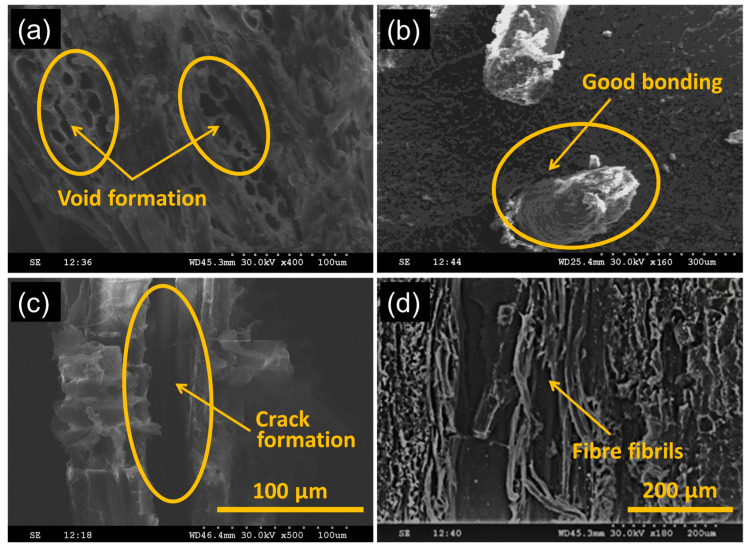
SEM images of composite that underwent cryogenic treatment for duration of (**a**) 30 min, (**b**) 60 min and (**c**) 90 min. (**d**) Formation of fibrils from fibres.

**Figure 4:**
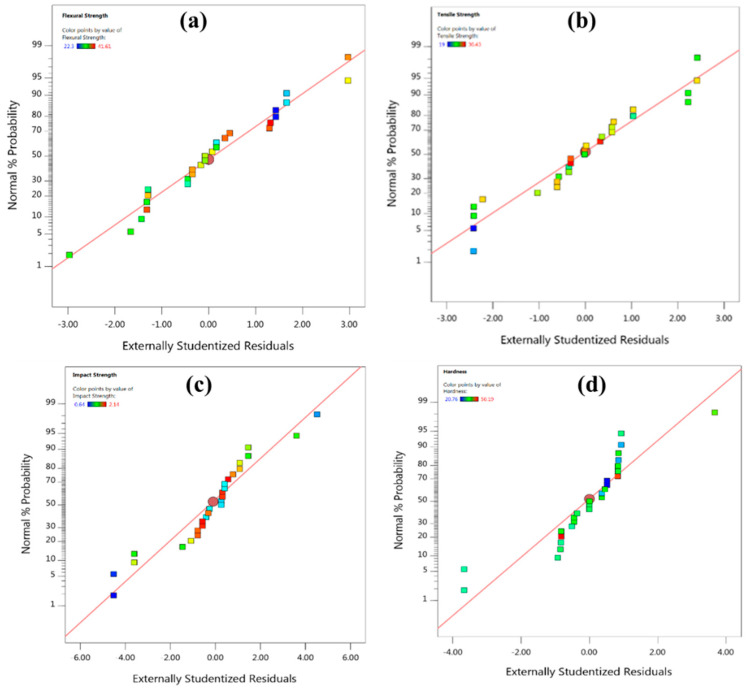
Normal probability plots for (**a**) flexural strength (**b**) tensile strength, (**c**) impact strength and (**d**) hardness.

It was observed that mechanical properties were better at 60 min of cryogenic treatment. Figure 3a–d show the fractured surface of composites with cryogenic treatment. The cryogenic treatment showed adverse effects in the composites leading to reduced strength. The composites experienced high internal stresses during the cryogenic treatment. The internal stress at the interface region affected the fibre and matrix bonding [24]. Further expanding the treatment length was found to diminish the mechanical properties as a result of the developments of break on the material because of the high internal stresses [25]. However, with the continued increase of the cryogenic treatment time, cracks developed at secluded regions because the fibres, saw-dust and the matrix constricted at different rates [26], Figure 4c. However, the composite with the alkaline-treated fibre showed good bonding (Figure 3b). The increased internal stress at the increased cryogenic treatment time, burst the fibres into fibrils (Figure 3d), and this negatively affected the stress distribution during loading and reduced the strength. The investigation by Liu et al. [27] demonstrated that the fibre under cryogenic conditions caused variations in the hemicellulose and lignin content, but not in the cellulose content. In comparison, it can be stated that the physical and chemical properties of the jute fibre in the composites may have changed after the cryogenic treatment, which may have influenced the composites’ strength.

### 3.2. Model Analysis and Evaluation

Cubic models (third order polynomial functions) were developed using Equation (3) for mechanical properties (tensile strength, flexural strength, impact strength and hardness) from the data recorded as per the experimental matrix designed based on Taguchi L_27_ orthogonal array as shown in Table 2. The developed models were then analysed for normality using normal probability plots. These plots were found to follow normal distribution as could be evidenced from Figure 4. Normal plot also serves as a diagnostic plot for the validity of analysis of variance (ANOVA).

**Figure 5:**
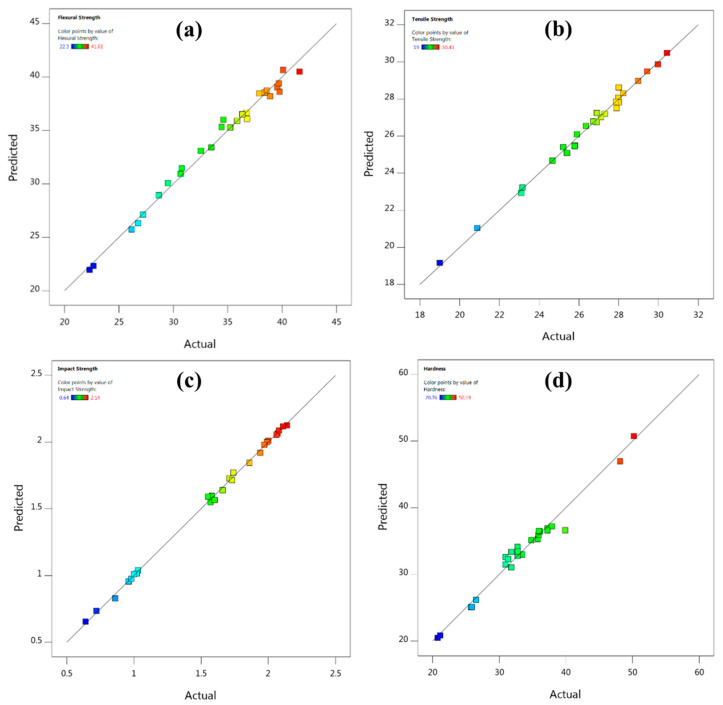
Actual vs. predicted values for (**a**) flexural strength (**b**) tensile strength, (**c**) impact strength and (**d**) hardness.

The predicted vs. actual plots for the properties in Figure 5 reveal the homogenous nature of the variance. The models were then evaluated (Table 3) for goodness of fit with actual values using coefficient of determination (R^2^). All the values were very close to 1, which indicate that the models have a good fit [23]. High values of Adjusted R^2^ closer to 1 also imply good accuracy of the developed models. Values of adequate precision for all property models were greater than 4, which indicate that they can be used to navigate the design space. Low values of coefficient of variation (CoV%) indicate that the reliability of the performed experiments was very high.

### 3.3. Flexural Strength

Only the factors and their two-way interactions that have a “significant effect” on mechanical properties (*p*-values < 0.05) were included in the ANOVA. For flexural strength in Table 4, the F-value (15.51) implies that the cubic model is significant, and there is only 0.82% possibility for this large F-value to occur due to errors. Other insignificant model terms were neglected. The number of jute layers and the duration of cryogenic treatment have a significant effect (*p*-values < 0.05) on the flexural strength of the fabricated composite material. Since flexural failures are dictated by the rupture of the cell walls in fibre bundles, the increase in number of jute layers has worked favourably in increasing the flexural strength of the composite [28]. The duration of cryogenic treatment also affected flexural strength significantly because of development of cracks in isolated regions as shown in SEM images in Figure 3c.

The final model equation for flexural strength in terms of coded factors is given below. This equation could be used to make predictions about flexural strength for the given levels of the factors. Their relative impact could be identified by comparing the coefficients of the factors.

Flexural Strength = +37.36 − 2.84 × A − 3.12 × B + 2.5 × C − 3.97 × D − 4.18 × E + 0.57 × A × B − 7.09 × A × C − 1.49 × A × E − 6.87 × B × C − 0.4825 × B × E + 1.91 × C × E + 0.5543 × D × E − 4.81 × E × F.

**Figure 6:**
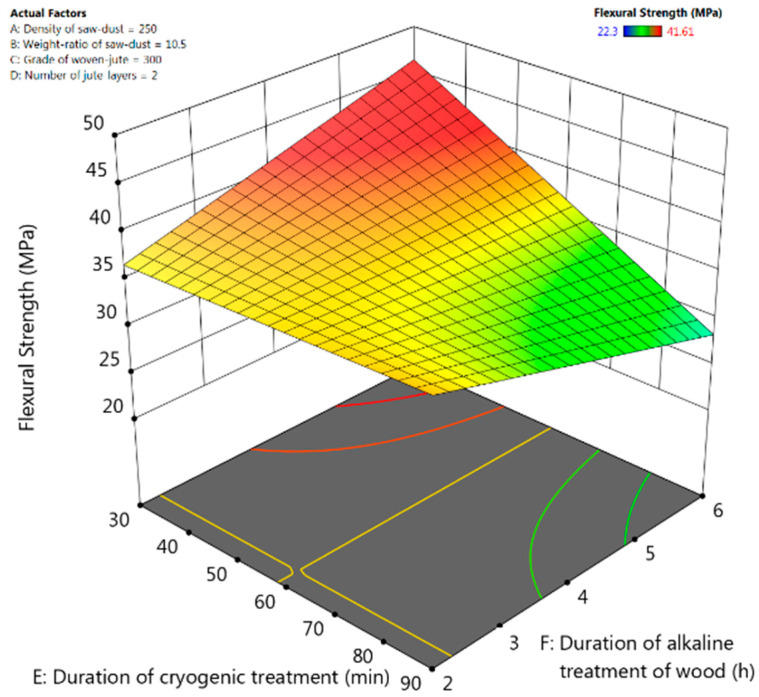
Response-surface plots for flexural strength for interactive effect between the duration of alkaline treatment and the duration of cryogenic treatment of composite.

From the interactive response-surface plots in Figure 6, it can be seen that the flexural strength is maximum in the region where the duration of cryogenic treatment is lower, and the duration of alkaline treatment is higher. Flexural strength was found to increase gradually with increase in duration of alkaline treatment of jute-fibre due to enhanced bonding between the matrix and fibre as seen from SEM images in Figure 3b.

### 3.4. Tensile Strength

**Figure 7:**
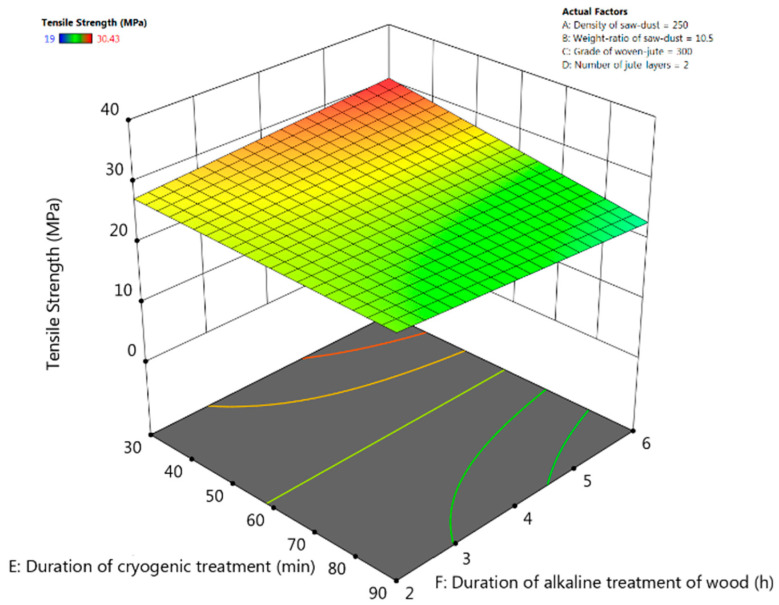
Response-surface plots for tensile strength for interactive effect between the duration of alkaline treatment and the duration of cryogenic treatment of composite.

The F-value (24.65) from Table 5 of ANOVA implies that the cubic model for tensile strength is significant and there is only 0.34% possibility for this large F-value to occur due to errors. The weight ratio of the saw-dust, number of jute layers and the duration of cryogenic treatment have significant effect on tensile strength of the composite (*p*-values < 0.05). From the interactive response-surface plots in Figure 7, it could be seen that the tensile strength is maximum in the region where the duration of cryogenic treatment is lower, and the duration of alkaline treatment is higher. Tensile strength was found to increase with increase in duration of alkaline treatment of jute-fibre when the duration of cryogenic treatment is kept lower. When the duration of cryogenic treatment was high, the tensile strength of the composite suffered due crack formation as witnessed in SEM image in Figure 3c. The external damage induced in the fibres during treatment was the main reason for the crack development. The externally damaged areas acted as the crack nucleating points in the matrix. This is supported by the investigation of Zhang et al. [29]. The final model equation for tensile strength in terms of coded factors is given below. This equation could be used to make predictions about flexural strength for the given levels of the factors.

Tensile Strength = +26.92 − 1.20 × A − 2.77 × B + 0.4014 × C − 2.45 × D − 2.19 × E - 0.21 × A × B − 5.64 × A × C + 0.1642 × A × E − 3.00 × B × C − 0.0900 × B × E − 0.0933 × C × E − 0.2967 × D × E − 1.64 × E × F.

### 3.5. Impact Strength

The F-value (132.16) from ANOVA in Table 6 implies that the cubic model for impact strength is significant and there is only 0.01% possibility for this large F-value to occur due to errors. The weight ratio of saw-dust, and the number of jute layers have a significant effect on the impact strength of the fabricated composite material (*p*-values < 0.05).

**Figure 8:**
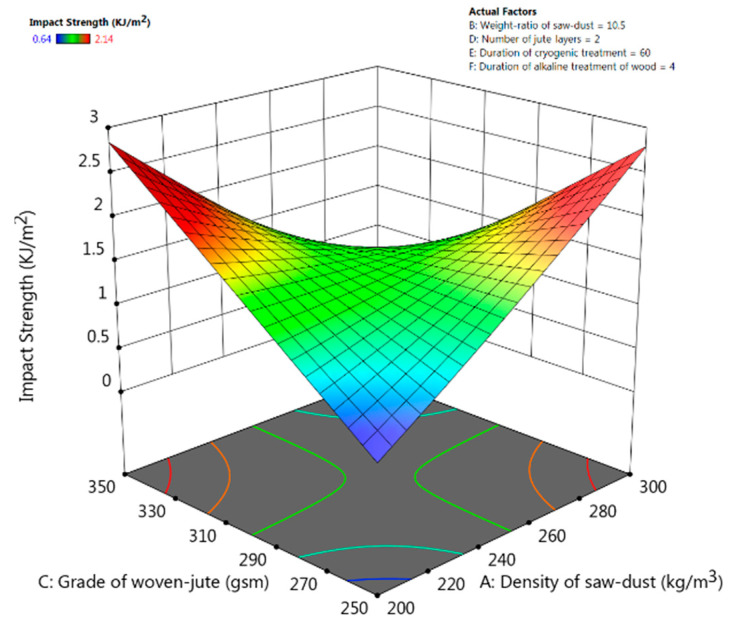
Response-surface plots for tensile strength for interactive effect between the density of saw-dust and the grade of woven-jute.

From the response-surface plots in Figure 8, it is clear that the impact strength of the composite will be optimum when there is a balance between the grade of woven-jute fibre and the density of saw-dust. Impact strength is higher either at regions of higher grades of jute-fibre and lower density of saw-dust or at regions of lower grades of jute-fibre and higher density of saw-dust filler. The final model equation for impact strength in terms of coded factors is given below. This equation could be used to make predictions about impact strength for the given levels of the factors.

Impact Strength = +1.52 − 0.0541 × A − 0.2204 × B - 0.0307 × C − 0.7059 × D − 0.0556 × E − 0.0917 × A × B − 1.23 × A × C − 0.0997 × A × E − 0.4367 × B × C − 0.0750 × B × E + 0.1019 × C × E − 0.294 × D × E − 0.0017 × E × F.

### 3.6. Hardness

**Figure 9:**
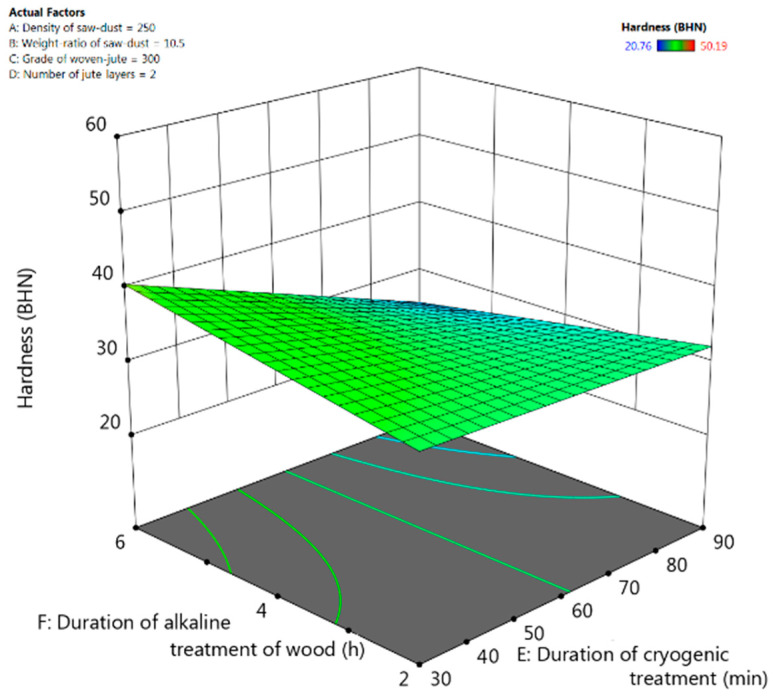
Response-surface plots for tensile strength for interactive effect between the duration of alkaline treatment and the duration of cryogenic treatment of composite.

The F-value (7.19) from Table 7 implies that the cubic model for hardness is significant and there is only 3.41% possibility for this large F-value to occur due to errors. The weight ratio of saw-dust and the duration of cryogenic treatment have a significant effect (*p*-values < 0.05) on the hardness of the fabricated composite material. From the interactive response-surface plots in Figure 9, it could be observed that higher hardness of the composite could be achieved by increasing the duration of alkaline treatment of jute fibres and by keeping duration of cryogenic treatment lower.

The final model equation for hardness in terms of coded factors is given below. This equation could be used to make predictions about hardness for the given levels of the factors.

Hardness = +32.91 − 0.7455 × A − 7.72 × B − 1.05 × C − 6.51 × D − 4.20 × E − 0.4467 × A × B − 15.23 × A × C + 2.53 × A × E − 7.15 × B × C − 1.78 × B × E − 4.79 × C × E − 3.64 × D × E − 3.52 × E × F

### 3.7. Optimisation

The criteria for optimisation with an objective to maximise the mechanical properties is shown in Table 8. Table 9 lists the two topmost solutions obtained by employing desirability approach using Equation (5). These solutions have higher desirability, which implies that they are closer to the set objective. From this approach, saw-dust of density 300 kg/m^3^ used as a filler with a weight ratio of 13% and a reinforcement of a single layer of woven-jute-fibre mat of grade 250 gsm subjected to alkaline treatment for 4 h in a composite that has undergone 45 min of cryogenic treatment delivers an impact strength of 3.3375 kJ/m^2^, flexural strength of 44.9604 MPa, tensile strength of 33.4353 MPa and hardness of 51.4875 BHN. During cryogenic treatment, fibre undergoes variation in its diameter and also in its chemical composition. This affects the interfacial bonding leading to lowering of the strength. This observation can be corroborated by the results of the investigation of Ma et al. [24].

### 3.8. Validation

The solutions from Table 9 generated using the desirability approach were validated by confirmatory trials. Three trials were taken for each combination and the readings were averaged. The final experimental values were compared with the solutions suggested by desirability approach. Table 10 presents the results of the confirmatory trials in conjunction with the solutions from Table 9. It was found that the experimental values were closer in agreement with the predicted values with error in prediction below 5%. This suggests that the models developed using RSM were able to describe the effect of the six parameters on the mechanical properties adequately. Table 11 shows the response values at optimum factor levels obtained using the desirability approach when compared to untreated woven-jute/saw-dust polyester composite with similar density of saw-dust, weight ratio of saw-dust, grade of woven-jute and number of jute layers.

It can be seen that composite with saw-dust of density 300 kg/m^3^ used as a filler with a weight ratio of 13% and a reinforcement of a single layer of woven-jute-fibre mat of grade 250 gsm subjected to alkaline treatment for 4 h in a composite that has undergone 45 min of cryogenic treatment presented an improvement of 64% in impact strength, 20.75% in flexural strength, 14.8% in tensile strength and 27.7% in hardness when compared to the composite reinforced with untreated fibres.

## 4. Conclusions and Future Perspectives

In this study, the effect of six parameters (density of saw-dust, weight ratio of saw-dust, grade of woven-jute, number of jute layers, duration of cryogenic treatment of composite and duration of alkaline treatment of jute-fibre) on mechanical properties of the composite was investigated to enhance maximise tensile strength, impact strength, flexural strength and hardness. A Taguchi L_27_ orthogonal array was used to design the experiments and Response-surface methodology (RSM) was used to model, predict and plot interactive surface plots. The desirability approach was employed to find the optimal combination of parameters. The following conclusions were deduced:

- The degree of adhesion quality between fibre reinforcement and the polymer matrix improved with alkaline treatment of the jute-fibres. However, mechanical properties were better only up to 60 min of cryogenic treatment beyond which cracks developed in isolated regions of the composite.
- It was found that the composite with saw-dust of density 300 kg/m^3^ used as a filler with a weight ratio of 13% and a reinforcement of a single layer of woven-jute-fibre mat of grade 250 gsm subjected to alkaline treatment for 4 h in a composite that has undergone 45 min of cryogenic treatment presented an improvement of 64% in impact strength, 21% in flexural strength, 15% in tensile strength and 28% in hardness when compared to the composite reinforced with untreated fibres.
- These results show that chemically treated fibre reinforcement could provide enhanced mechanical properties at cryogenic environment, thus the composites developed with treated fibres can be effective for the use in the structural application in cold environments. Such composites are also applicable for the use in space crafts and fuel storage applications. The future work in this area could focus on investigating hybrid synthetic and natural fibre-based composites’ performance in the cryogenic environment. This would create a balance between enhanced properties as well as sustainability.

## Figures and Tables

**Table 1:** Parameters and their levels.

No.	Constraints	Symbol	Stages		
			L_1_	L_2_	L_3_
1	Teak Wood density (kg/m^3^)	A	200	250	300
2	Teak Wood weight ratio (%)	B	6	12	15
3	Woven Jute type (gsm)	C	250	300	350
4	Number of jute layer’s (no)	D	1	2	3
5	Cryogenic treatment (min)	E	30	60	90
6	Alkaline treatment (h)	F	2	4	6

**Table 2:** Experimental design matrix based on L_27_ orthogonal array.

Run	A: Density of Saw-Dust (kg/m^3^)	B: Weight Ratio of Saw-Dust (%)	C: Grade of Woven-Jute (gsm)	D: Number of Jute Layers	E: Duration of Cryogenic Treatment (min)	F: Duration of Alkaline Treatment (h)
1	200	6	250	1	30	2
2	200	6	250	1	60	4
3	200	6	250	1	90	6
4	200	12	300	2	30	2
5	200	12	300	2	60	4
6	200	12	300	2	90	6
7	200	15	600	3	30	2
8	200	15	600	3	60	4
9	200	15	600	3	90	6
10	250	6	300	3	30	4
11	250	6	300	3	60	6
12	250	6	300	3	90	2
13	250	12	600	1	30	4
14	250	12	600	1	60	6
15	250	12	600	1	90	2
16	250	15	250	2	30	4
17	250	15	250	2	60	6
18	250	15	250	2	90	2
19	300	6	600	2	30	6
20	300	6	600	2	60	2
21	300	6	600	2	90	4
22	300	12	250	3	30	6
23	300	12	250	3	60	2
24	300	12	250	3	90	4
25	300	15	300	1	30	6
26	300	15	300	1	60	2
27	300	15	300	1	90	4

**Table 3:** Model evaluation.

Parameters	Flexural Strength (MPa)	Tensile Strength (MPa)	Impact Strength (kJ/m^2^)	Hardness (BHN)
R^2^	0.9884	0.9927	0.9986	0.9753
Adj. R^2^	0.9247	0.9524	0.9911	0.8396
Adeq. Precision	13.3507	21.5391	34.5107	12.4844
CoV%	4.48	2.15	3.00	7.87

**Table 4:** Results of ANOVA for flexural strength including only the significant parameters.

Source	Sum of Squares	Mean Square	F-Value	*p*-Value
Model	785.68	35.71	15.51	0.0082
D-Number of jute layers	18.43	18.43	8.00	0.0474
E-Duration of cryogenic treatment	74.22	74.22	32.23	0.0048
AC	32.31	32.31	14.03	0.0200
BC	38.57	38.57	16.75	0.0149
EF	136.32	136.32	59.19	0.0015
Residual	9.21	2.30		
Cor Total	794.89			

**Table 5:** Results of ANOVA for tensile strength including only the significant parameters.

Source	Sum of Squares	Mean Square	F-Value	*p*-Value
Model	175.76	7.99	24.65	0.0034
B-Weight ratio of saw-dust	12.68	12.68	39.12	0.0033
D-Number of jute layers	7.04	7.04	21.72	0.0096
E-Duration of cryogenic treatment	20.33	20.33	62.72	0.0014
AC	20.47	20.47	63.15	0.0014
BC	7.35	7.35	22.69	0.0089
EF	15.76	15.76	48.64	0.0022
Residual	1.30	0.3241		
Correlation Total	177.05			

**Table 6:** Results of ANOVA for impact strength including only the significant parameters.

Source	SS	Mean Square	F-Value	*p*-Value
Model	6.19	0.2815	132.16	<0.0001
B-Weight ratio of saw-dust	0.0800	0.0800	37.54	0.0036
D-Number of jute layers	0.5831	0.5831	273.73	<0.0001
AC	0.9779	0.9779	459.03	<0.0001
BC	0.1560	0.1560	73.23	0.0010
Residual	0.0085	0.0021		
Correlation Total	6.20			

**Table 7:** Results of ANOVA for hardness including only the significant parameters.

Source	Sum of Squares	Mean Square	F-Value	*p*-Value
Model	1091.13	49.60	7.19	0.0341
B-Weight ratio of saw-dust	98.15	98.15	14.22	0.0196
E-Duration of cryogenic treatment	74.77	74.77	10.84	0.0302
AC	149.20	149.20	21.62	0.0097
EF	73.20	73.20	10.61	0.0312
Residual	27.60	6.90		
Correlation Total	1118.73			

**Table 8:** The criteria for optimisation with an objective to maximise the mechanical properties. A weighting of 1 indicates more emphasis to the objective, while 0.1 indicates less emphasis.

Factors	Target	Limits		Weight	
		Lower	Upper	Lower	Upper
A: Density of saw-dust (kg/m^3^)	is in range	200	300	1	1
B: Weight ratio of saw-dust	is in range	6	15	1	1
C: Grade of woven-jute (gsm)	is in range	250	350	1	1
D: Number of jute layers	is in range	1	3	1	1
E: Duration of cryogenic treatment (min)	is in range	30	90	1	1
F: Duration of alkaline treatment of wood (h)	is in range	2	6	1	1
**Responses**	**Objective**	**Lower**	**Upper**	**Lower**	**Upper**
Flexural Strength (MPa)	maximise	22.3	41.61	0.1	1
Tensile Strength (MPa)	maximise	19	30.43	0.1	1
Impact Strength (KJ/m^2^)	maximise	0.64	2.14	0.1	1
Hardness (BHN)	maximise	20.76	50.19	0.1	1

**Table 9:** Predicted solutions by desirability approach closer to objectives (maximised conditions).

No.	A	B	C	D	E	F	Impact Strength	Flexural Strength	Tensile Strength	Hardness
	kg/m^3^	%	gsm	No	min	h	KJ/m^2^	MPa	MPa	BHN
1	300	13	250	1	45	4	3.3375	44.9604	33.4353	51.4875
2	300	15	250	1	64	3	2.9591	43.3230	32.0891	52.6094

**Table 10:** Confirmatory test results with % error (maximised conditions).

No.	A	B	C	D	E	F		Impact Strength	Flexural Strength	Tensile Strength	Hardness
	kg/m^3^	%	gsm	No	min	h		kJ/m^2^	MPa	MPa	BHN
1	300	13	250	1	45	4.0	Predicted	3.3375	44.9604	33.4353	51.4875
							Actual	3.2040	43.0720	34.7392	53.9074
							% Error	4.0	4.2	3.9	4.7
2	300	15	250	1	64	3.0	Predicted	2.9591	43.323	32.0891	52.6094
							Actual	3.0715	45.0559	33.1801	50.3471
							% Error	3.8	4.0	3.4	4.3

**Table 11:** Optimised response in comparison with untreated woven-jute/saw-dust polyester composite (maximised conditions).

No.	A	B	C	D	E	F	Impact Strength	Flexural Strength	Tensile Strength	Hardness
	kg/m^3^	%	gsm	No	min	hr	kJ/m^2^	MPa	MPa	BHN
Untreated	300	15	250	1	0	0	2.0350	37.2331	29.1245	40.3265
Treated	300	13	250	1	45	4	3.3375	44.9604	33.4353	51.4875
Improvement (%)							64.0049	20.7538	14.8012	27.6765

## Data Availability

Not applicable.

## Supplementary Materials

The following are available online at https://www.mdpi.com/article/10.3390/polym13152471/s1.

## References

[1] Salman S.D. (2020). Effects of jute fibre content on the mechanical and dynamic mechanical properties of the composites in structural applications. Def. Technol..

[2] Ganesan V., Kaliyamoorthy B. (2020). Utilization of Taguchi Technique to Enhance the Interlaminar Shear Strength of Wood Dust Filled Woven Jute Fiber Reinforced Polyester Composites in Cryogenic Environment. J. Nat. Fibers.

[3] Velmurugan G., Babu K. (2020). Statistical analysis of mechanical properties of wood dust filled Jute fiber based hybrid composites under cryogenic atmosphere using Grey-Taguchi method. Mater. Res. Express.

[4] Velmurugan G., Babu K., Flavia L.I., Stephy C.S., Hariharan M. (2020). Utilization of grey Taguchi method to optimize the mechanical properties of hemp and coconut shell powder hybrid composites under liquid nitrogen conditions. IOP Conf. Ser. Mater. Sci. Eng..

[5] Townsend T., Sette J., Fangueiro R., Rana S. (2016). Natural Fibres and the World Economy. Natural Fibres: Advances in Science and Technology Towards Industrial Applications.

[6] Swain P.T.R. (2013). Physical and Mechanical Behavior of Al2O3 Filled Jute Fiber Reinforced Epoxy Composites. Int. J. Curr. Eng. Technol..

[7] Saha A.K., Das S., Bhatta D., Mitra B.C. (1999). Study of jute fiber reinforced polyester composites by dynamic mechanical analysis. J. Appl. Polym. Sci..

[8] Munikenche Gowda T., Naidu A.C.B., Chhaya R. (1999). Some mechanical properties of untreated jute fabric-reinforced polyester composites. Compos. Part A Appl. Sci. Manuf..

[9] Vinod A., Vijay R., Singaravelu D.L. (2018). ThermoMechanical Characterization of *Calotropis gigantea* Stem Powder-Filled Jute Fiber-Reinforced Epoxy Composites. J. Nat. Fibers.

[10] Tavassoli F., Razzaghi-Kashani M., Mohebby B. (2018). Hydrothermally treated wood as reinforcing filler for natural rubber bio-composites. J. Polym. Res..

[11] Pinto M.A., Chalivendra V.B., Kim Y.K., Lewis A.F. (2014). Evaluation of surface treatment and fabrication methods for jute fiber/epoxy laminar composites. Polym. Compos..

[12] De Assis F.S., Pereira A.C., da Costa Garcia Filho F., Lima É.P., Monteiro S.N., Weber R.P. (2018). Performance of jute non-woven mat reinforced polyester matrix composite in multilayered armor. J. Mater. Res. Technol..

[13] Park J.-M., Quang S.T., Hwang B.-S., DeVries K.L. (2006). Interfacial evaluation of modified Jute and Hemp fibers/polypropylene (PP)-maleic anhydride polypropylene copolymers (PP-MAPP) composites using micromechanical technique and nondestructive acoustic emission. Compos. Sci. Technol..

[14] Rafiquzzaman M., Islam M., Rahman H., Talukdar S., Hasan N. (2016). Mechanical property evaluation of glass-jute fiber reinforced polymer composites: Glass-Jute Fiber Polymer Composites. Polym. Adv. Technol..

[15] Li X., Tabil L.G., Panigrahi S. (2007). Chemical Treatments of Natural Fiber for Use in Natural Fiber-Reinforced Composites: A Review. J. Polym. Environ..

[16] Ray D., Sarkar B.K., Rana A.K., Bose N.R. (2001). Effect of alkali treated jute fibres on composite properties. Bull. Mater. Sci..

[17] Rajesh G., Prasad A.V.R. (2014). Tensile Properties of Successive Alkali Treated Short Jute Fiber Reinforced PLA Composites. Procedia Mater. Sci..

[18] Sanjeevi S., Shanmugam V., Kumar S., Ganesan V., Sas G., Johnson D.J., Shanmugam M., Ayyanar A., Naresh K., Neisiany R.E. (2021). Effects of water absorption on the mechanical properties of hybrid natural fibre/phenol formaldehyde composites. Sci. Rep..

[19] Vigneshwaran S., Sundarakannan R., John K.M., Johnson R.D.J., Prasath K.A., Ajith S., Arumugaprabu V., Uthayakumar M. (2020). Recent advancement in the natural fiber polymer composites: A comprehensive review. J. Clean. Prod..

[20] Ning N., Fu S., Zhang W., Chen F., Wang K., Deng H., Zhang Q., Fu Q. (2012). Realizing the enhancement of interfacial interaction in semicrystalline polymer/filler composites via interfacial crystallization. Prog. Polym. Sci..

[21] Ku H., Wang H., Pattarachaiyakoop N., Trada M. (2011). A review on the tensile properties of natural fiber reinforced polymer composites. Compos. Part B Eng..

[22] Owen M.M. (2014). Original Article The effects of alkali treatment on the mechanical properties of jute fabric reinforced epoxy composites. Int. J. Fiber Text. Res..

[23] Vera Candioti L., De Zan M.M., Cámara M.S., Goicoechea H.C. (2014). Experimental design and multiple response optimization. Using the desirability function in analytical methods development. Talanta.

[24] Ma H.-L., Jia Z., Lau K.-T., Leng J., Hui D. (2016). Impact properties of glass fiber/epoxy composites at cryogenic environment. Compos. Part B Eng..

[25] Wu Z., Li J., Huang C., Li L. (2015). Effect of Matrix Modification on Interlaminar Shear Strength of Glass Fibre Reinforced Epoxy Composites at Cryogenic Temperature. Phys. Procedia.

[26] Surendra Kumar M., Chawla N., Priyadarsini A., Mishra I., Ray B.C. (2007). Assessment of Microstructural Integrity of Glass/Epoxy Composites at Liquid Nitrogen Temperature. J. Reinf. Plast. Compos..

[27] Liu J., Guan Z., Li Z. (2018). Application of cryogenic and mechanical treatment in degumming of hemp stems. Biosyst. Eng..

[28] Kulkarni A.G., Satyanarayana K.G., Sukumaran K., Rohatgi P.K. (1981). Mechanical behaviour of coir fibres under tensile load. J. Mater. Sci..

[29] Zhang Y., Xu F., Zhang C., Wang J., Jia Z., Hui D., Qiu Y. (2016). Tensile and interfacial properties of polyacrylonitrile-based carbon fiber after different cryogenic treated condition. Compos. Part B Eng..

